# Testicular Rupture or Testicular Fracture? A Case Report and Literature Review

**DOI:** 10.1155/2018/1323780

**Published:** 2018-11-14

**Authors:** Firas Addas, Sylvia Yan, Marios Hadjipavlou, Michael Gonsalves, Samer Sabbagh

**Affiliations:** ^1^Department of Urology, St George's Hospital, London, UK; ^2^Department of Radiology, St George's Hospital London, UK

## Abstract

Testicular trauma is relatively uncommon. However, severe injuries can result in many complications and should be carefully diagnosed and managed. We present a case of testicular fracture diagnosis made by ultrasonography. The surgical exploration revealed the fracture as well as complete rupture of the tunica albuginea. Testicular rupture is the disruption of the tunica albuginea, while testicular fracture is a “break” in the testicular parenchyma. Management could be conservative in mild fracture cases without rupture while suspected or confirmed fracture should be treated by surgical exploration.

## 1. Introduction

Testicular trauma is relatively uncommon as the organs are protected by the dependent and mobile nature of the scrotum. However, severe testicular injury can result in organ loss which can affect fertility contribute to hypogonadism and affect social confidence. Rupture or fracture of the testis might result in the release of spermatic antigens and the subsequent exposure to the immune system resulting in the production of antisperm antibodies. This might lead to infertility by affecting sperm function [1], and early surgical intervention within 72 hours is necessary to prevent these complications [2]. In this paper we present a case of testicular injury and we discuss the difference between testicular rupture and testicular fracture by reviewing the literature on the terminology.

## 2. Case Presentation

A 32-year-old motorcyclist was admitted following a collision with a car travelling at 30 mph. His scrotum impacted the motorbike handlebars and he was thrown over the car bonnet. He complained of right-sided testicular pain. The past medical history was uneventful. Clinical examination revealed normal lie of the right testicle with a small contained haematoma. The left testicle was entirely normal. Abdominal examination was unremarkable. The patient was cleared of all nonurological injuries.

**Figure 1:**
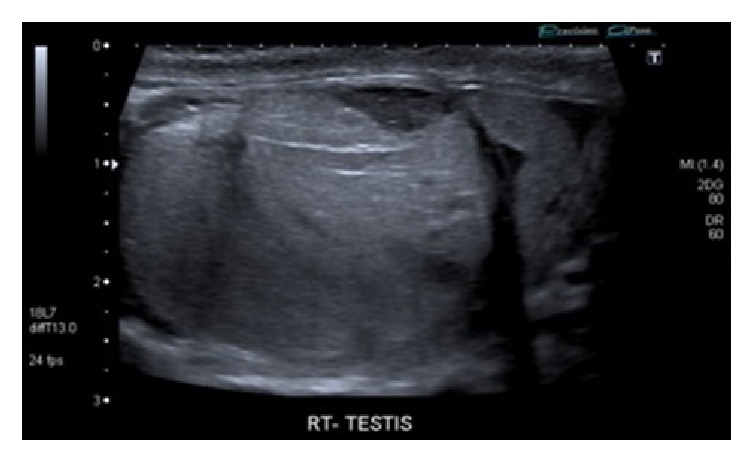
Fractured right testicle on ultrasound scan.

**Figure 2:**
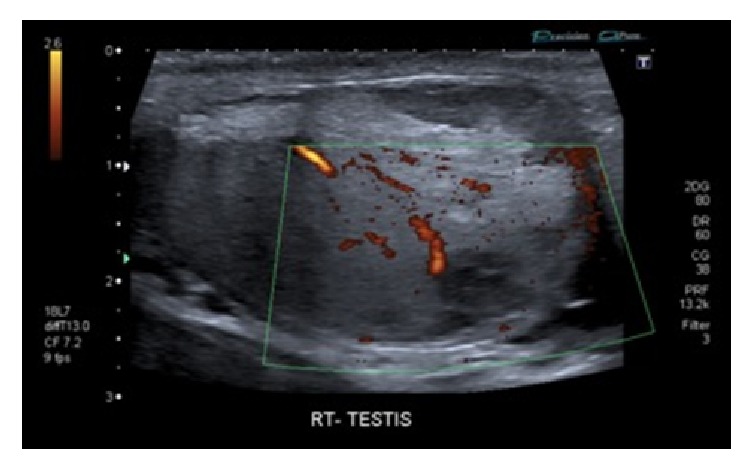
Fractured right testicle with Doppler flow to some areas on ultrasound scan.

Scrotal ultrasonography showed irregular hypoechoic regions in the lower pole of the right testis with no significant Doppler flow evident. The tunica albuginea appeared intact and there was slightly increased Doppler flow in the rest of the testis. The left testis was normal. (Figures 1 and 2). The ultrasound scan findings suggested a right testicular fracture to be the most likely pathology but rupture could not be excluded or confirmed. Both testes appeared normal in size.

A decision for surgical exploration was made and through a midline scrotal incision, the right haematocoele was evacuated. The lower pole of the right testis was seen to be fractured in a bi-valve pattern. The tunica albuginea was circumferentially dismembered (Ruptured) and the testicle was seen as nearly two separate lobes. As both halves appeared largely viable, it was decided to spare the testis and reconstruct. An area of necrotic tissue was excised, after which the tunica albuginea was closed with 4/0 vicryl continuous suture. There injury to the tail of the epididymis was also repaired. Histopathology of the excised testicular tissue confirmed haemorrhage and focal necrosis.

**Figure 3:**
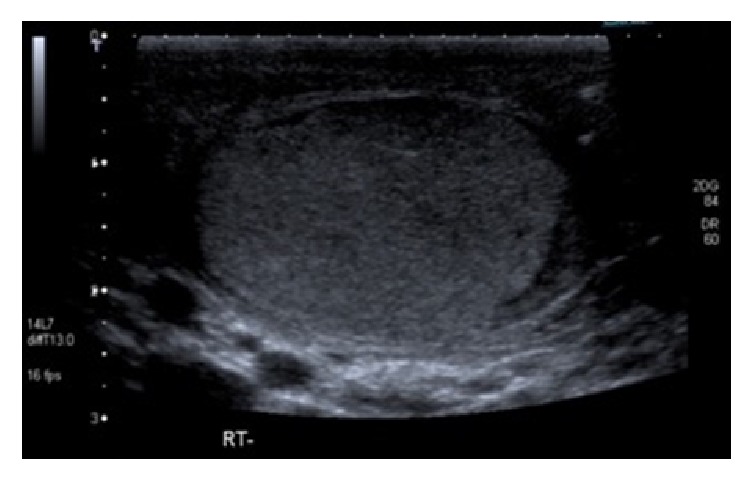
Follow-up ultrasound scan of right testis with a small amount of residual haematoma in scrotal sac.

**Figure 4:**
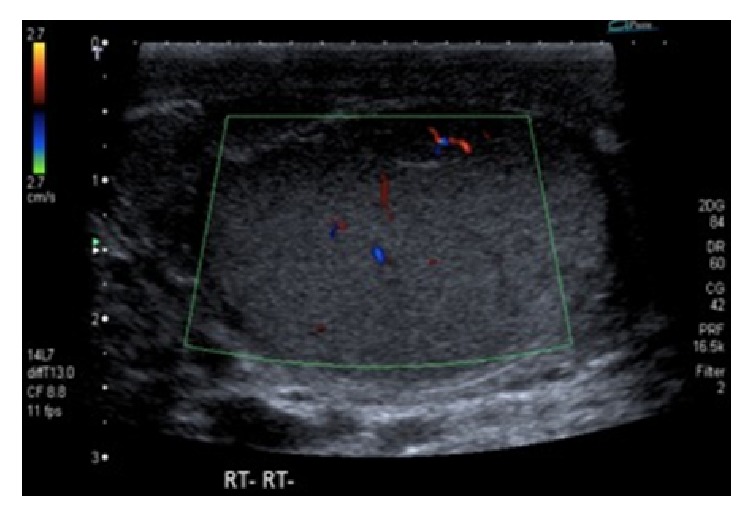
Follow-up ultrasound scan of right testis with normal perfusion.

A follow-up ultrasound scan testes at one month revealed an entirely normal testicle (Figures 3 and 4). The patient made an uneventful recovery and he was discharged after two months.

## 3. Discussion

Blunt injury accounts for most of testicular trauma cases and it occurs most commonly during sporting injuries, straddle injuries, motor vehicle accidents, and assaults, mostly affecting males aged between 10 and 30 years [3, 4]. A detailed history and physical examination are important to reach an accurate diagnosis. High frequency ultrasound with a linear array transducer is the modality of choice for investigating suspected testicular trauma. When compared to surgical findings, the sensitivity and specificity of ultrasonography in blunt testicular trauma are 100% and 93.5%, respectively [5].

The tunica albuginea is a tough, fibrous outer covering of the testis. Testicular rupture is defined as interruption of the tunica albuginea (or change in contour) or extrusion of seminiferous tubules [6]. Testicular rupture typically occurs when the testis is forced against the inferior pubic ramus or the pubic symphysis as a result of rapid deceleration and an applied force of 50 kg has been shown to be sufficient for testicular rupture [7, 8]. The normal tunica albuginea in ultrasonography appears as two parallel hyperechoic layers outlining the testis; therefore a discontinuity of the tunica albuginea with history of scrotal trauma directs to a diagnosis of testicular rupture [9].

A retrospective study by Dalton et al found that the discontinuity of the echogenic tunica albuginea is the main sign of testicular rupture which indicates the necessity of surgical intervention [10]. Sonographic features of testicular rupture include heterogeneous echotexture within the testis, testicular contour abnormality, and disruption of the tunica albuginea [5, 11].

Another paper used the absence of normal vascularity within the testis as an indicator that may help characterize a rupture [9].

An abnormality of the contour of the testis is considered indirect evidence of testicular rupture in the case of a large extratesticular haematocoele or a large scrotal wall hematoma that may obscure the site of tunical disruption [9]. Due to the close apposition of the tunica vasculosa to the tunica albugenia, testicular rupture is associated with disruption of both structures in the majority of cases. As a result, rupture of the testis results in a loss of vascularity to a portion or the entirety of the testis, depending on the grade of the injury [9].

Testicular fracture refers to a break or discontinuity in the normal testicular parenchyma. It is diagnosed using ultrasonography and appears as a linear hypoechoic and avascular area within the testis. This finding can be associated with rupture of the tunica albuginea. Only 17% of cases demonstrate a fracture line [12–15].

In such cases, color Doppler imaging plays an important role in directing management [9]. The presence of vascularity within the testicular parenchyma is indicative of its salvageability. In many instances, debridement alone along the line of fracture is necessary, while the vascular parenchyma is preserved [11].

In our case, the patient was reported to have a fracture of the testis on ultrasonography; however on exploration the testis was completely ruptured. In these cases, magnetic resonance imaging does have higher sensitivity and a diagnostic accuracy of 100% [16]. However due to the severity of the case the decision to operate was made. This case underlines the importance of urgent surgical exploration, even in cases of an ultrasound finding of testicular fracture, when the clinical suspicion of testicular rupture is high. Careful inspection of the scrotal contents is of paramount importance, irrespective of the imaging findings. Evacuation of the haematoma, tissue debridement and closure of any defects should be performed if necessary. It this case, the decision to repair and salvage the testis was correct as the organ subsequently made a full recovery.

In patients with testicular trauma, ultrasonography performed by an experienced operator can yield useful information to guide management. Accurate terminology should be used when reporting the findings—testicular rupture is the disruption of the tunica albuginea, while testicular fracture is a “break” in the testicular parenchyma. The two findings can occur in synchrony. Management should always be tailored to the specific injuries; however urgent scrotal exploration should be the gold standard in investigating and treating suspected testicular rupture.
